# Ep-CAM expression in squamous cell carcinoma of the esophagus: a potential therapeutic target and prognostic marker

**DOI:** 10.1186/1471-2407-6-165

**Published:** 2006-06-23

**Authors:** Nikolas H Stoecklein, Annika Siegmund, Peter Scheunemann, Andreas M Luebke, Andreas Erbersdobler, Pablo E Verde, Claus F Eisenberger, Matthias Peiper, Alexander Rehders, Jan Schulte am Esch, Wolfram Trudo Knoefel, Stefan B Hosch

**Affiliations:** 1 Klinik für Allgemein- und Viszeralchirurgie, Universitätsklinikum Düsseldorf, D-40225 Düsseldorf, Germany; 2 Klinik für Allgemein-, Viszeral- und Thoraxchirugie, Universitätsklinikum Hamburg-Eppendorf, D-20246 Hamburg, Germany; 3 Institut für Pathologie, Universitätsklinikum Hamburg-Eppendorf, D-20246 Hamburg, Germany; 4 Koordinierungszentrum für klinische Studien, Universitätsklinikum Düsseldorf, D-40225 Düsseldorf, Germany; 5 Chirurgische Klinik, Universitätsklinikum Hamburg-Eppendorf, D-20246 Hamburg, Germany

## Abstract

**Background:**

To evaluate the expression and test the clinical significance of the epithelial cellular adhesion molecule (Ep-CAM) in esophageal squamous cell carcinoma (SCC) to check the suitability of esophageal SCC patients for Ep-CAM directed targeted therapies.

**Methods:**

The Ep-CAM expression was immunohistochemically investigated in 70 primary esophageal SCCs using the monoclonal antibody Ber-EP4. For the interpretation of the staining results, we used a standardized scoring system ranging from 0 to 3+. The survival analysis was calculated from 53 patients without distant metastasis, with R0 resection and at least 2 months of clinical follow-up.

**Results:**

Ep-CAM neo-expression was observed in 79% of the tumors with three expression levels, 1+ (26%), 2+ (11%) and 3+ (41%). Heterogeneous expression was observed at all expression levels. Interestingly, tumors with 3+ Ep-CAM expression conferred a significantly decreased median relapse-free survival period (log rank, p = 0.0001) and median overall survival (log rank, p = 0.0003). Multivariate survival analysis disclosed Ep-CAM 3+ expression as independent prognostic factor.

**Conclusion:**

Our results suggest Ep-CAM as an attractive molecule for targeted therapy in esophageal SCC. Considering the discontenting results of the current adjuvant concepts for esophageal SCC patients, Ep-CAM might provide a promising target for an adjuvant immunotherapeutic intervention.

## Background

Advances in surgical techniques over the last decade improved the outcome of patients with squamous cell carcinomas (SCC) of the esophagus significantly. However, in comparison to other gastrointestinal malignancies, esophageal SCC belongs to the more aggressive tumors with 5-year survival rates averaging below 30 per cent [1,2]. From the survival data of patients receiving surgery with curative intention it is obvious, that at the time of initial diagnosis in most patients unperceived tumor cell spread has occurred. The results of current multimodal adjuvant and neoadjuvant strategies for esophageal SCC to eliminate the minimal residual tumorload are still unsatisfactory and due to their unspecificity afflicted with significant side effects [3-5]. Therefore new adjuvant therapeutic concepts are urgently needed to eradicate effectively the minimal residual disease and to improve the post-operative prognosis of esophageal SCC patients.

A promising basis for new systemic anti-cancer therapy represents the epithelial cellular adhesion molecule Ep-CAM, encoded by the 9-exon gene *TACSTD1 *[6,7] (Ep-CAM, EGP 40, GA733-2, 17-1A) that was recently re-mapped to chromosome 2p21 [8]. EpCAM is a 40 kD type I transmembrane glycoprotein with two epidermal growth factor like repeats in the external domain and a short intracellular domain consisting of two α-actin binding sites for actin cytoskeleton linkage and functions as an intercellular adhesion molecule modulating cadherin-mediated adhesions and thereby adhesion strength [9-12]. The physiologic expression of Ep-CAM in adult human tissues is strictly restricted to the basolateral cell membrane of glandular, pseudo-stratified and transitional epithelia, whereas normal squamous stratified epithelia are Ep-CAM negative [13]. Interestingly, de novo expression of Ep-CAM occurs during squamous cell carcinogenesis of the oral cavity and of the lung[14]. The expression level increases during the progression from mild dysplasia to carcinoma [14]. Although the biological role of Ep-CAM in healthy tissues and in cancer is not understood conclusively, its overexpression is observed in several cancer types and has been associated with poor prognosis in breast cancer [15,16] and gallbladder cancer [17]. Of much interest, from the clinical point of view, is the possibility to use Ep-CAM as a target for immunotherapy [18-21]. So far, very few data are available regarding Ep-CAM expression in esophageal cancer. Here we investigated the expression and prognostic impact of Ep-CAM in esophageal SCC to test the potential value of this molecule for antibody based adjuvant therapy in this aggressive cancer.

## Methods

The ethics committee of the chamber of physicians of Hamburg approved this study. Informed consent was obtained from all patients before inclusion into the study. Tumor samples were collected from 70 patients with resectable esophageal carcinoma who had undergone radical en bloc esophagectomy at the University Hospital Hamburg Eppendorf, Germany. Tumor stage and grade were classified by the routine histopathologic assessment according to the UICC (Union Internationale Contre le Cancer) Classification for Malignant Tumors [22,23] from pathologists unaware of the immunohistochemical findings. The survival analysis was calculated from 53 patients with R0 resection, where at least two months of prospectively evaluated clinical follow-up was available. Seventeen patients were excluded from the survival analysis because of metastatic disease (n = 5), perioperative death (n = 5), non-tumor free resection margins (n = 5) and lost for follow-up (n = 2). The clinico-pathologic data are presented in Table 1 (all patients) and in Table 2 (the 53 patients included in the survival analysis).

### Tissue preparation and immunohistochemistry

The tumor tissue was snap-frozen in liquid nitrogen immediately after removal and stored at -80°C until use. The Ep-CAM antigen was detected with the monoclonal antibody Ber-EP4 (IgG1, Dako, Hamburg, Germany), which can be used on snap frozen material as described previously[24]. Briefly, 5 μm cryostat sections were cut from each tumor and were transferred onto glass slides pretreated with 3-triethoxysilyl-propylamin (Merck, Darmstadt, Germany). One section was stained with conventional hematoxylin an eosin (H & E) staining and the following section was stained using the alkaline phosphatase-antialkaline phosphatase (APAAP) technique[24]. Sections of normal colonic mucosa and MCF-7 cells served as positive staining controls, and isotype-matched, irrelevant murine monoclonal antibodies served as negative controls (MOPC 21 for IgG1; Sigma, Deisenhofen, Germany). For the evaluation of the Ep-CAM staining we used standardized criteria to evaluate membranous staining specified by Dako for interpretation of the HercepTest for p185^HER2 ^expression. The Dako scoring system has a scale from 0 - 3+: 0, no staining or 10% or less of the tumor cells show any level of positive staining; 1+, a faint membrane staining is detected in more than 10% of the tumor cells or the cells are only stained in part of their membrane; 2+, weak to moderate staining of the entire membrane is observed in more than 10% of the tumor cells; 3+, strong staining of the entire membrane in more than 10% of the tumor cells. Heterogeneous staining was defined as different staining intensity in more than 25% of Ep-CAM-positive tumor cells. The inter-observer reproducibility in our study was 93%. The slides with discrepant assessments were reevaluated, and a consensus was reached in all cases.

### Statistical analysis

To test the correlation between the clinico-pathological data and the level of Ep-CAM expression we used the Fisher's exact test and whenever appropriate the χ^2^-test. All of the variables were dichotomised. For analysis of follow-up data, life table curves were calculated using the Kaplan-Meier method, and survival distributions were compared using the log-rank test. The primary end points were disease-specific survival or relapse-free survival, as measured from the date of surgery to the time of the last follow-up or cancer-related death or tumor relapse, respectively. Data of patients who were still alive and without evidence of tumor relapse at the end of the study were censored. For multivariate analysis, a parametric survival regression model based on a Weibull distribution was applied. Model selection was carried out using the Bayesian Information Criterion (BIC) [25,26]. This information criterion was adopted to search for a parsimonious model that fit the data [27]. The lower the BIC the better the model explains the data. A search in the model space was done with a stepwise algorithm. The range of the models examined in the stepwise search was from the simplest model with a constant term, to the most complex model with all covariates included. The investigated distributions were loglogistic, lognormal, logistic, exponential, Gaussian and Weibull. Both, the exponential and the Weibull distributions fitted the data best. Because the exponential distribution is a particular case of the Weibull when the scale parameter equals one, we decided to use the Weibull distribution. Statistical computations were done with SPSS and the statistical package R [28]. The level of significance was set at p < 0.05.

## Results

### Expression of Ep-CAM in normal esophagus and esophageal SCC

**Figure 1:**
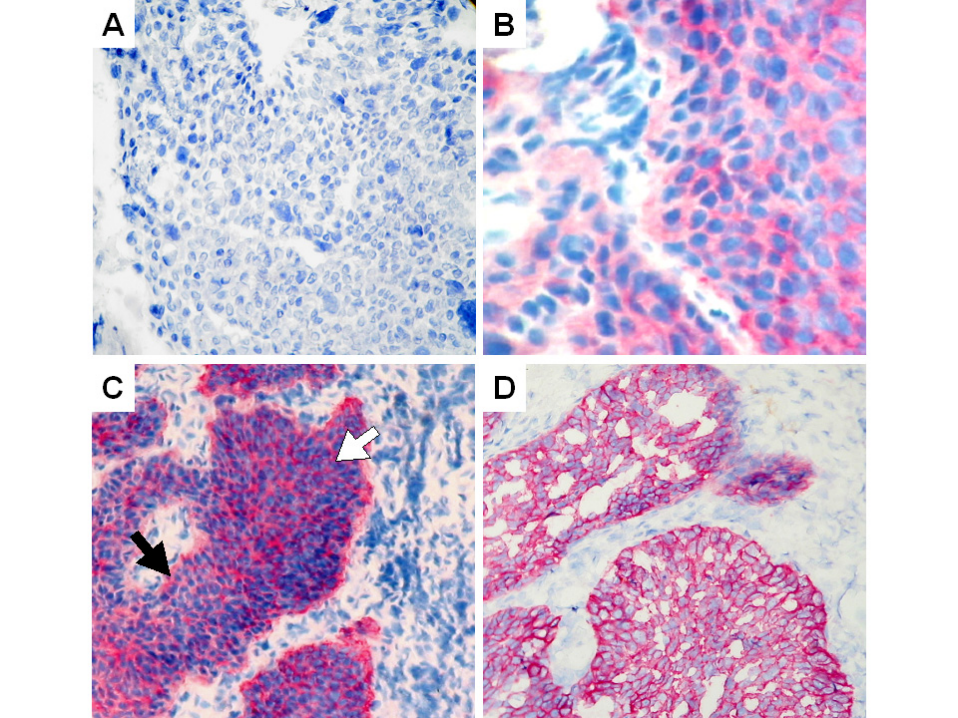
**Ep-CAM expression in esophageal SCC**. *A*, esophageal SCC without Ep-CAM neo-expression. *B*, Detail of a tumor with 2+ Ep-CAM expression. *C*, Tumor with heterogeneous 3+ Ep-CAM expression (white arrow: strong expression; black arrow: faint expression) and D, shows a representative sample with homogenous 3+ Ep-CAM expression.

In a first step, we investigated the Ep-CAM expression in 10 normal, non-pathologic squamous epithelia of the esophagus. As previously reported [13], the non-pathologic esophageal squamous epithelium was negative for Ep-CAM expression. In contrast four different levels of Ep-CAM expression (Figure 1), which were classified according to the Dako scoring system, were observed in the 70 esophageal SCC. We found 3+ Ep-CAM expression in 29/70 of the investigated tumors (41%). Eight of the 70 samples were scored as 2+ (11%) and their membranous Ep-CAM staining was less intense, when compared to the 3+ samples. A weak Ep-CAM staining scored as 1+ was observed in 18/70 (26%) tumors and an absent expression of Ep-CAM was detected in 15/70 tumors (21%). Neither in the complete patient collective (n = 70) nor in the group with clinical follow up (n = 53, Table 1 and 2, respectively) any statistical significant correlation between the level of Ep-CAM expression and the clinico-pathological parameters pT-category, pN-category and grading were observed. It is important to note that a heterogeneous Ep-CAM expression was seen at all expression levels and no correlation was observed between Ep-CAM heterogeneity within a tumor and its Ep-CAM expression score (Chi-square test, p = 0.134). Also, a specific staining pattern, e.g. intense staining at the invasive front, was not detected.

### Prognostic influence of Ep-CAM expression in esophageal SCC

**Figure 2:**
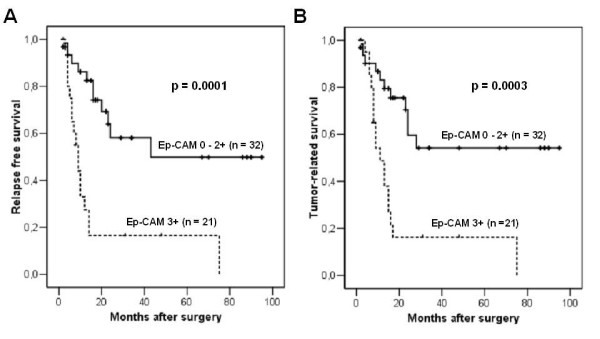
**Prognostic impact of Ep-CAM**. Kaplan-Meier curves for relapse-free (*A*) and overall survival (*B*) calculated from 53 patients with esophageal SCC.

In the 53 patients with follow-up data and a median observation time of 15.0 months (range: 2 – 95 months) we found, that the general neo-expression of Ep-CAM (1+ - 3+) did not correlate with the patient's prognosis. The median cumulative disease-specific survival of the patients was 17.0 months for Ep-CAM 1+ - 3+ positive tumors and 24.0 months for Ep-CAM negative tumors (log rank, p = 0.3491; relapse-free survival: Ep-CAM 1+ - 3+ 16.0 months vs. Ep-CAM negative 43.0 months; log rank, p = 0.5993). Interestingly, tumors exhibiting a strong Ep-CAM expression (3+) conferred a significantly decreased median relapse-free survival interval. The median cumulative relapse-free survival interval of patients with Ep-CAM 3+ expression was 9.0 months, compared to 43.0 months for patients with 0 - 2+ Ep-CAM expression (log rank, p = 0.0001) (Figure 2A). The median disease-specific survival was 11.0 months for patients with Ep-CAM 3+ expressing tumors compared to > 24.0 months for those with 0-2+ Ep-CAM staining (log rank, p = 0.0003, Figure 2B). As expected, the pN-category was also of prognostic significance in our investigated series with a median disease-specific survival of 13.0 months for patients with positive nodal status compared to 75.0 months for patients without regional lymph node involvement (log rank, p = 0.0170). The prognostic value of the pT-category for disease-specific survival was not statistically significant (pT1–2 vs. pT 3–4, 16 months vs. 28 months, respectively; log rank, p = 0.1178). Tumor grading was also of no prognostic significance.

For multivariate regression analysis we included Ep-CAM expression (Ep-CAM 3+ vs. Ep-CAM 0-2+), sex, age, pN, pT and grading. We found an independent prognostic influence for Ep-CAM 3+ on relapse-free survival and disease-specific survival (p = 0.002 and p = 0.005, respectively) (Table 3). We also tested the possible interaction between the pN category and Ep-CAM expression within a multivariate model and confirmed the indepence of both, Ep-CAM and pN-category as independent prognostic factors (data not shown).

## Discussion

Here we present the first comprehensive study of Ep-CAM expression in primary esophageal SCC and its impact on prognosis. In our study, almost 80% of the primary esophageal SCC showed de novo expression of Ep-CAM within the primary tumor, while normal squamous mucosa was negative for Ep-CAM expression. To quantify the level of Ep-CAM expression we used the evaluation criteria for the FDA approved HercepTest (Dako) for quantification of the *ERBB2 *(HER2) gene product p185, the target for the therapeutic antibody trastuzumab (Herceptin). We used these guidelines because, comparable to p185, Ep-CAM is a membranous protein, and the criteria are standardized and are already used to assign patients to antibody-based therapy. The benefit or special applicability of the Hercep-Test Scoring System to evaluate EpCAM staining is so far unproven and needs to be confirmed in independent future studies. However, applying this scoring system, we identified three different levels of Ep-CAM expression. Interestingly, strong (3+) Ep-CAM expression was of prognostic significance. A correlation of strong Ep-CAM expression and poor prognosis has been also observed in breast cancer [15,16] and in gallbladder cancer [17]. In SCC of the lung, strong Ep-CAM expression was positively correlated to lymph node metastasis and larger tumors, however a correlation to survival could not been demonstrated [29].

Several lines of evidence point to the importance of this molecule in the early phase of squamous cell cancer progression. First, Ep-CAM de novo expression was already noted in weak, mild and severe dysplasias of squamous epithelium [14]. Second, this protein is expressed in the early phase of tumor cell dissemination and metastasis in esophageal and lung SCC, respectively. Ep-CAM expression is used to identify single tumor cells that have disseminated to lymph nodes and are associated with a poor prognosis [24,30,31]. Their malignant genotype was recently disclosed by single cell comparative genomic hybridization [32] and our group was able to demonstrate the proliferative capacity and tumorigenicity of these cells [33].

So far, the exact mechanisms of action of Ep-CAM contributing to the malignant potential of tumor cells are not fully understood. However, recently *in vivo *experiments gave the first proof of a direct link between Ep-CAM and cell cycle control. Münz et al. were able to show that upon Ep-CAM overexpression c-myc is rapidly upregulated [34]. Ep-CAM overexpression resulted in decreased growth factor requirement, enhanced metabolic activity and colony formation capacity. Importantly, expression of Ep-CAM anti-sense mRNA reversed these actions and led to a strong decrease in proliferation and metabolism. However, it is necessary to note in this context that functions and actions of Ep-CAM in cancer progression may differ in different cancer types. Although Ep-CAM expression is low in normal gastric mucosa and an increasing expression was observed in intestinal metaplasia, loss of Ep-CAM expression is a strong prognostic factor for poor survival in gastric cancer [35]. In renal cancer, loss of Ep-CAM expression was correlated with larger tumors and the presence of metastases. A positive Ep-CAM expression of the renal tumor cells was an independent prognostic marker for improved survival [36]. The biology behind these observations can be explained by *in vitro *and *in vivo *experiments demonstrating reduced motility, invasiveness and metastatic capability of Ep-CAM transfected cells [9,11,37]. These opposed findings in the different tumor types suggest a complex role of the Ep-CAM molecule, which functions might be regulated by the different predominant histogenetic molecular pathways.

Since in esophageal SCC Ep-CAM is overexpressed, its expression is associated with poor prognosis and its oncogenic potential *in vitro *render Ep-CAM an attractive molecule for adjuvant targeted therapy in this cancer. As a matter of fact, in patients with Dukes C colon cancer the administration of edrecolomab, a monoclonal murine IgG1 antibody directed against Ep-CAM, following the surgical tumor resection significantly improved overall survival by reducing the risk of tumor recurrence [38]. But this murine antibody rapidly looses its efficiency in humans due to neutralization by anti-edrecolomab antibodies [39] and other multicenter studies did not find beneficial effects on patients survival [40,41]. However, meanwhile, a fully humanized anti-Ep-CAM antibody (MT201) was developed and its efficacy of antigen dependent cellular cytotoxicity is by two orders of magnitude higher than that of edrecolomab [39]. More recently, Schlereth et al. reported about the very high antitumor efficacy of a bispecific single-chain antibody directed against Ep-CAM and CD3, which enables the efficient redirection and activation of tumor-resident T-cells [42].

Although we are aware, that our results of Ep-CAM expression in 70 esophageal SCC are somewhat preliminary and must be confirmed by a larger independent tumor collective, they are the first indication for a role of Ep-CAM in esophageal SCC progression. Neo-expression of Ep-CAM is a frequent event in this cancer and strong expression defines a subgroup with high risk for tumor relapse after complete tumor removal. Thus, Ep-CAM testing and application of therapeutic anti-EpCAM antibodies to suitable candidates represents a chance to improve the prognosis of esophageal SCC patients. This is particularly suggested in the adjuvant situation, since the population of minimal residual tumor cells is considered to be more amenable to antibody-based therapies, compared to large tumour masses in advanced stages of cancer [43].

## Conclusion

In conclusion, our results suggest Ep-CAM as an attractive molecule for targeted therapy in esophageal SCC, especially in the adjuvant situation. Considering the discontenting results of the current adjuvant concepts for esophageal SCC patients, anti-body based therapy directed against Ep-CAM holds promise for more effective eradication of minimal residual cancer to suppress lethal metastatic disease.

## Competing interests

The author(s) declare that they have no competing interests.

## Authors' contributions

NHS, SBH and WTK designed and coordinated the study, interpreted the data and wrote the manuscript. AS, AML and NHS performed and evaluated the immunohistochemical analysis. AS, PS and AR contributed substantially to the data acquisition and drafting the manuscript. AE was responsible for the histopathological evaluation, reviewed the cases and revised the manuscript critically. PEV contributed to and supervised the statistical analysis. CFE was involved in the sample acquisition, sample selection, clinical data acquisition and preparing the manuscript, MP and JSAE contributed to the acquisition and interpretation of the data and revised the manuscript critically. All authors read and approved the final version of the manuscript.

## Pre-publication history

The pre-publication history for this paper can be accessed here:



## Figures and Tables

**Table 1:** Patient and tumor characteristics (The numbers in parenthesis are percentages).*

		Ep-CAM expression			
Variable	Patients	0	1+	2+	3+

Patients		15	18	8	29
Male	50 (71)	10 (67)	12 (67)	5 (38)	23 (79)
Female	20 (29)	5 (33)	6 (33)	3 (62)	6 (21)
Primary Tumor					
pT1	15 (21)	4 (27)	3 (17)	3 (38)	5 (17)
pT2	11 (16)	3 (20)	2 (11)	2 (24)	4 (14)
pT3	42 (60)	8 (53)	11 (61)	3 (38)	20 (69)
pT4	2 (3)	0 (0)	2 (11)	0 (0)	0 (0)
Lymph node status					
pN0	33 (47)	7 (47)	9 (50)	6 (75)	11 (38)
pN1	37 (53)	8 (53)	9 (50)	2 (25)	18 (62)
Tumor grade					
G2	47 (67)	10 (67)	12 (67)	6 (75)	19 (66)
G3	23 (33)	5 (33)	6 (33)	2 (25)	10 (34)

*No statistical significant correlations between Ep-CAM expression and the histopathological parameters were observed.

**Table 2:** Patient and tumor characteristics of the 53 patients with clinical follow-up data (The numbers in parenthesis are percentages).*

		Ep-CAM expression			
Variable	Patients	0	1+	2+	3+

Patients		10	15	7	21
Male	39 (74)	8 (80)	10 (67)	4 (57)	17 (81)
Female	14 (26)	2 (20)	5 (33)	3 (43)	4 (19)
Primary Tumor					
pT1	15 (28)	4 (40)	3 (20)	3 (42)	5 (24)
pT2	8 (15)	1 (10)	2 (13)	2 (29)	3 (14)
pT3	30 (57)	5 (50)	10 (67)	2 (29)	13 (62)
Lymph node status					
pN0	27 (51)	4 (40)	9 (60)	5 (71)	9 (43)
pN1	26 (49)	6 (60)	6 (40)	2 (29)	12 (57)
Tumor grade					
G2	38 (72)	7 (70)	11 (73)	5 (71)	15 (71)
G3	15 (28)	3 (30)	4 (27)	2 (29)	6 (29)

*No statistical significant correlations between Ep-CAM expression and histopathological parameters were observed.

**Table 3:** Multivariate survival analysis for relapse-free and tumor-related survival of patients with esophageal SCC after best model selection by BIC.

Variable	Relative risk of death (CI 95%)	p-value
Relapse-free survival		
Lymph node metastasis Present vs. absent	3.068 (1.434 – 5.950)	< 0.001
Ep-CAM expression 3+ vs. 0 - 2+	5.392 (1.831 – 15.878)	< 0.001
Tumor-related survival		
Lymph node metastasis Present vs. absent	2.924 (1.577 – 5.421)	< 0.001
Ep-CAM expression 3+ vs. 0 - 2+	3.762 (1.471 – 9.619)	< 0.001
